# Supplementary figures and images for: Dengue in Cambodia 2002–2020: Cases, Characteristics and Capture by National Surveillance
Source: medRxiv. 2023 Apr 28:2023.04.27.23289207. Preprint. [Version 1] doi: 10.1101/2023.04.27.23289207 (PMC10274987; doi:10.1101/2023.04.27.23289207)

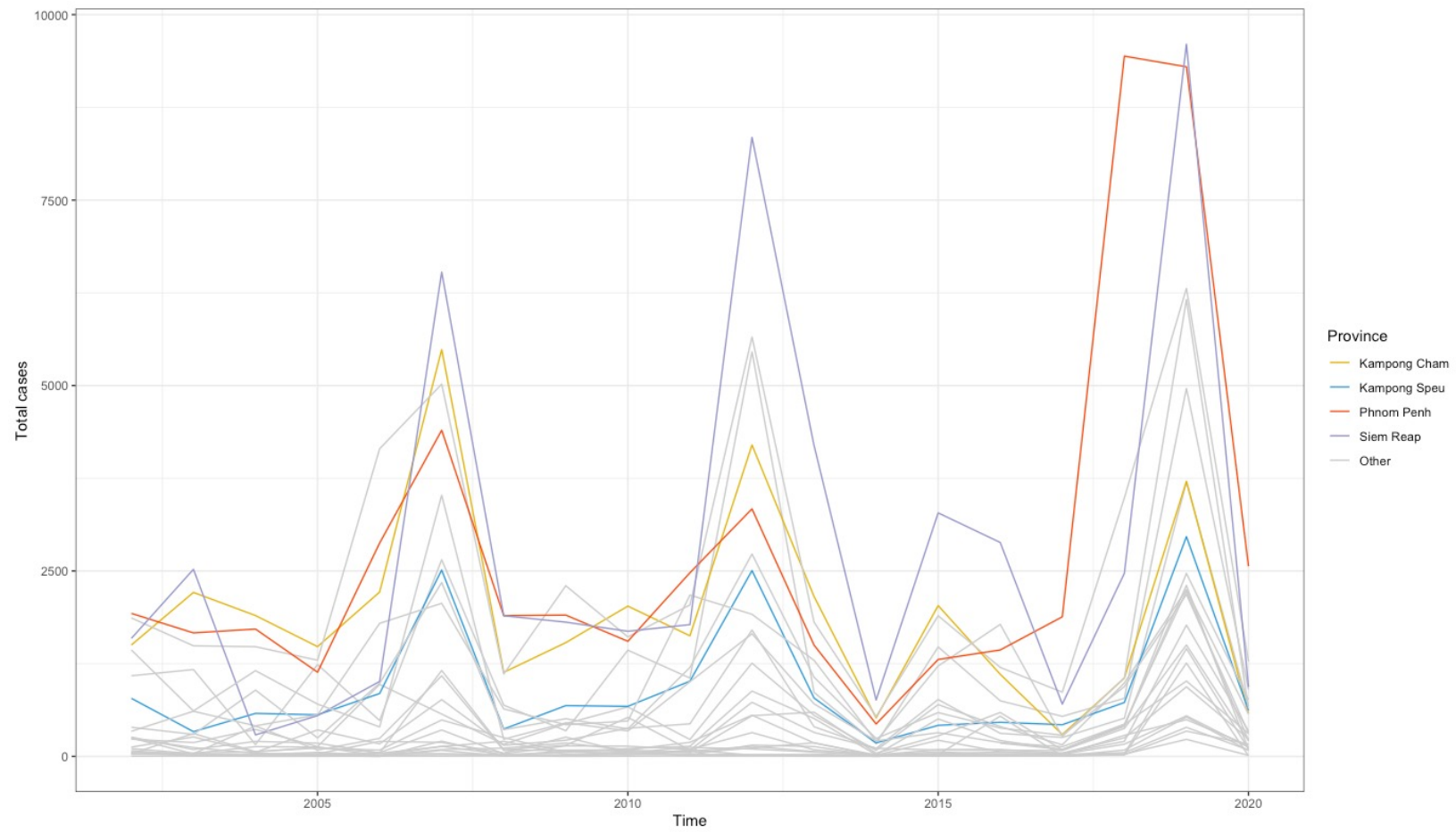

Supplement: Supplement 2 [file media-2.pdf]

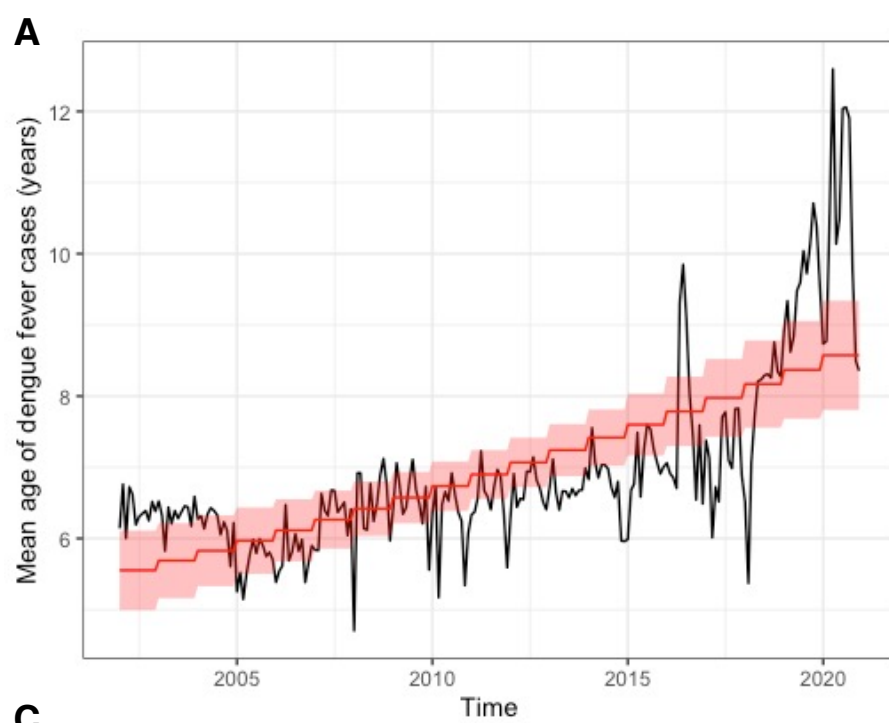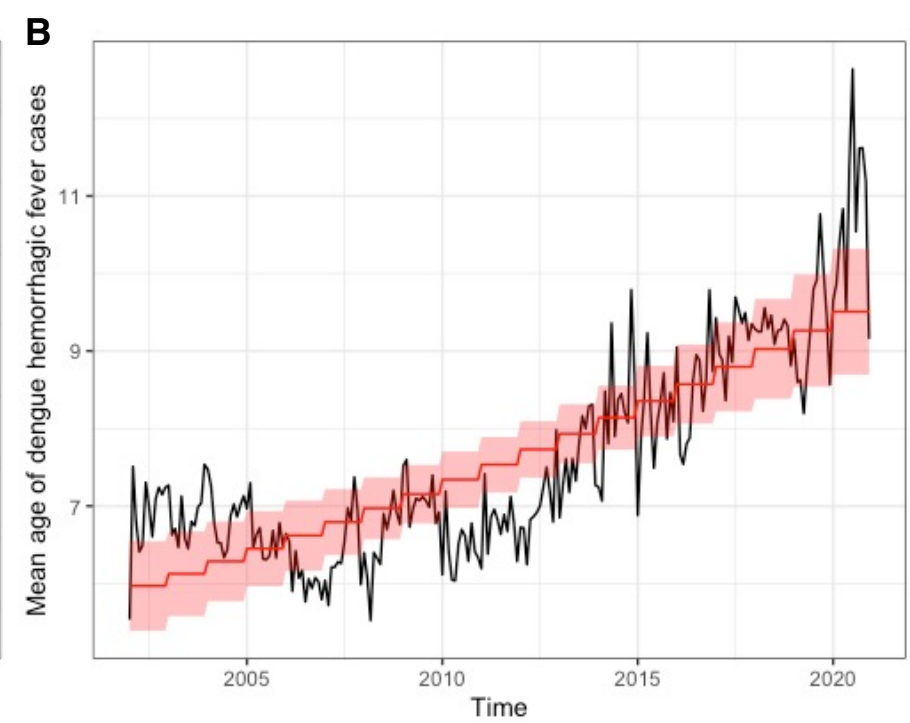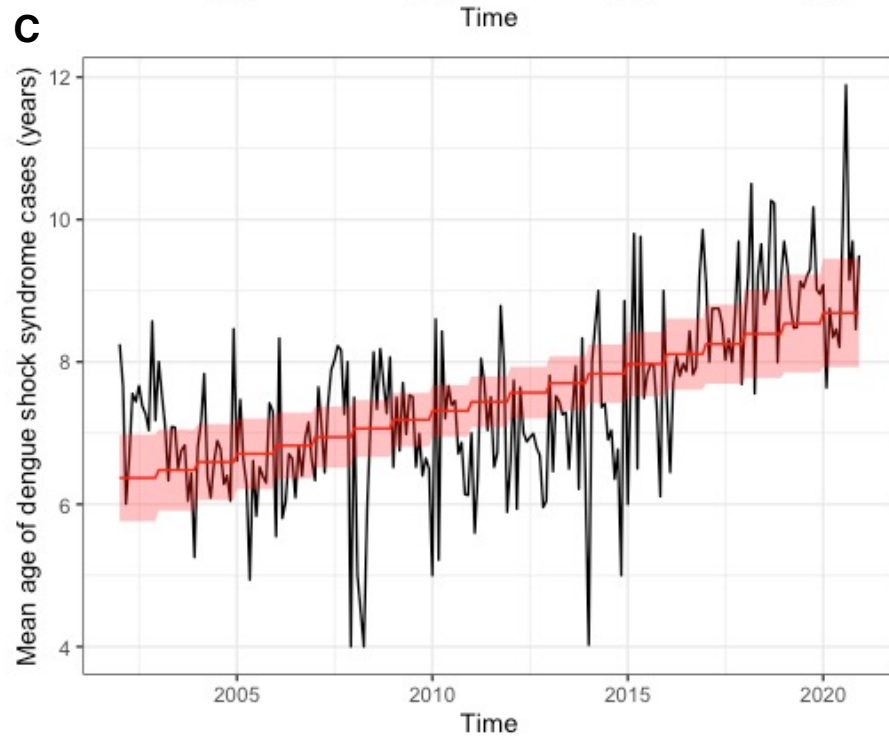

Supplement: Supplement 3 [file media-3.pdf]

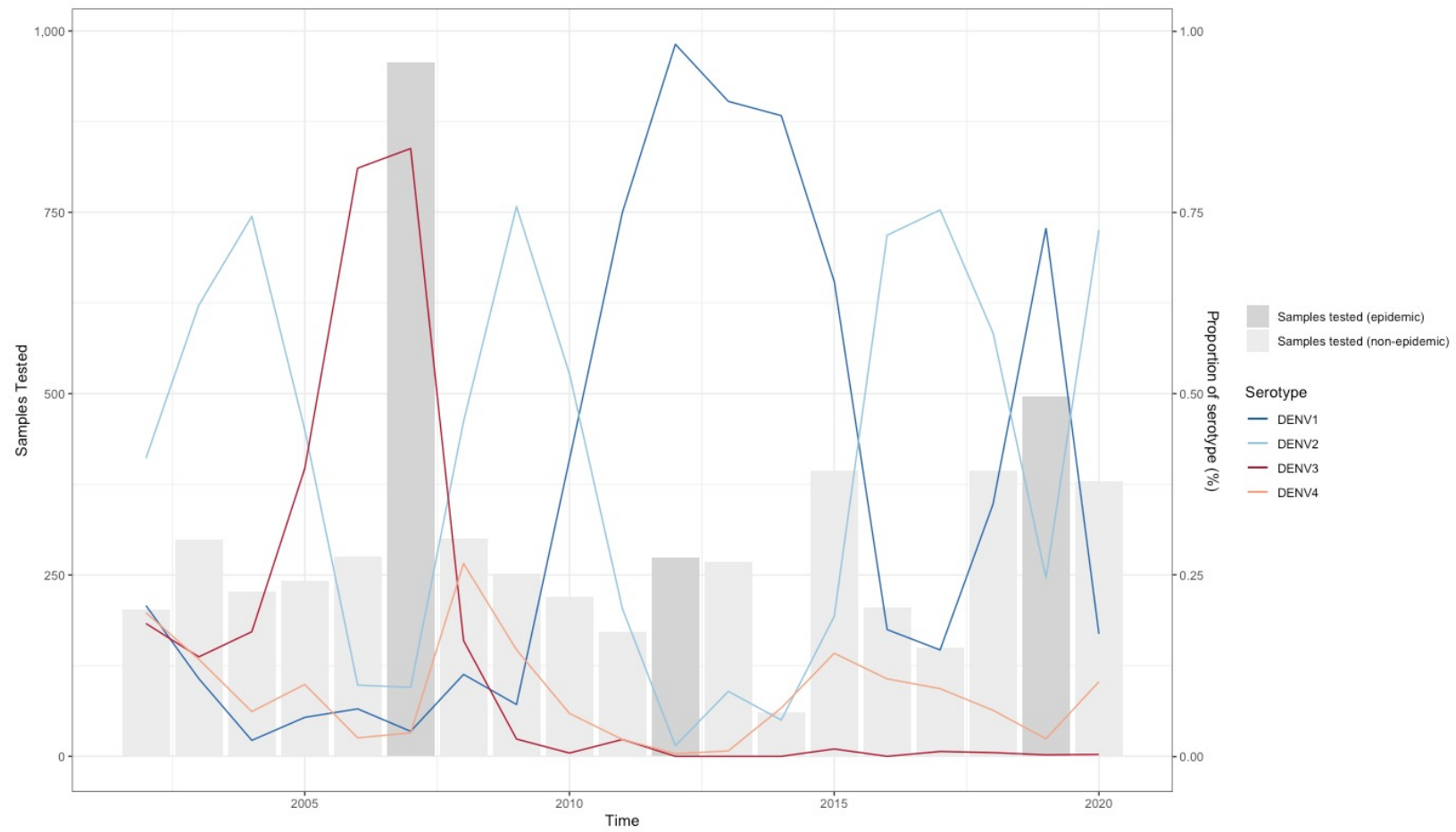

Supplement: Supplement 4 [file media-4.pdf]
